# An extraordinary rare presentation of liver hydatidosis with hydatid cyst scolices

**DOI:** 10.1002/ccr3.3111

**Published:** 2020-07-31

**Authors:** Spyridon Davakis, Athanasios Syllaios, Eleandros Kyros, Nikolaos Garmpis, Alexandros Charalabopoulos

**Affiliations:** ^1^ Laiko General Hospital National and Kapodistrian University of Athens Athens Greece; ^2^ Department of General Surgery Broomfield Hospital Mid Essex Hospital Services NHS Trust Chelmsford UK

**Keywords:** hooklets, hydatosis, liver, microscopic examination, scolices

## Abstract

The extraordinary finding of scolices with the characteristic hooklets may be found during the microscopic analysis in patients with cystic echinococcosis.

## Graphical abstract

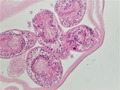

## 1 INTRODUCTION

Cystic echinococcosis is an endemic helminthic disease, caused by the infection of Echinoccocus granulosus metacestodes. Hereby, we present the case of a 59‐year‐old female patient with hydatid liver disease, who underwent cystectomy. The extraordinary finding of scolices with the characteristic hooklets of about 20 μm long was revealed during the microscopic analysis.

## 2 CLINICAL IMAGE

**Figure 1:**
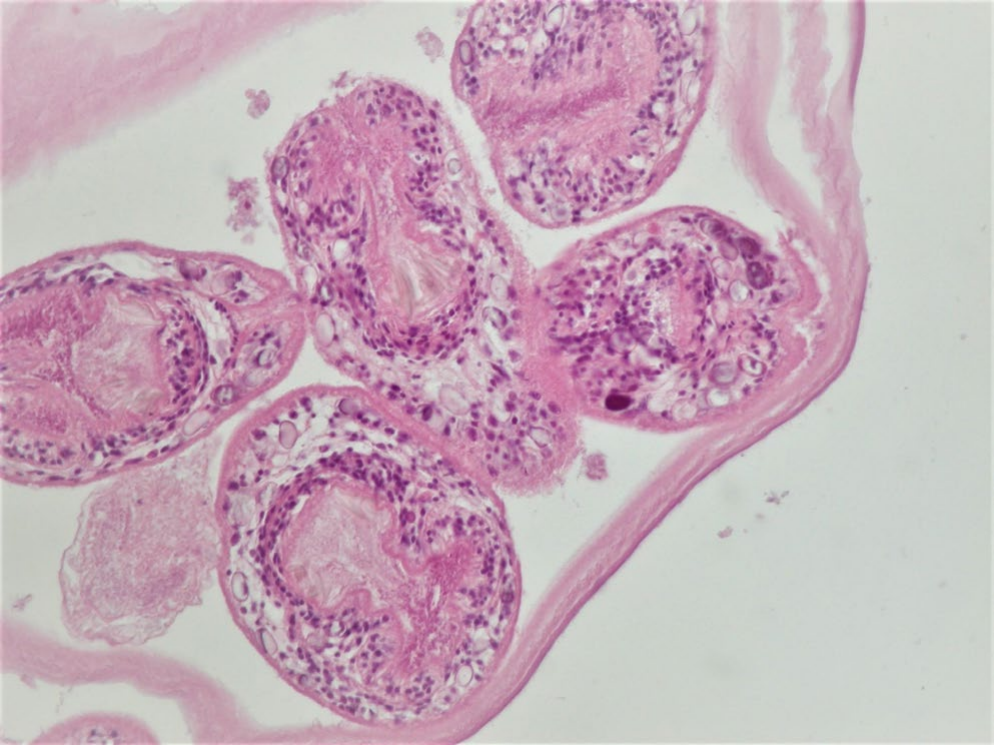
Scolices with the characteristic hooklets of about 20 μm long, microscopic examination (H&E ×100)

A 59‐year‐old Caucasian woman of Greek origin presented to our Department with a 2‐month history of right upper quadrant abdominal pain. Physical examination revealed mild abdominal tenderness during deep upper quadrant palpation. Total leukocyte count was 6400 uL^‐1^ with 1% eosinophils, and the positive serum antigen along with the imaging studies (Ultrasound, Computed Tomography) confirmed liver hydatidosis. Cystic echinococcosis is an endemic helminthic disease, caused by the infection of Echinoccocus granulosus metacestodes.^1^ Albendazole was given 4 weeks prior to surgery. During laparotomy, the clear liquid fluid that filled the cyst and the multiple daughter cysts were excised from liver segment VI. An extremely interesting finding was noted during microscopic examination. An extraordinary image, rarely seen nowadays in the Western world,^2^ of scolices with the characteristic hooklets of about 20 μm long was observed (Figure 1). Albendazole was administered postoperatively according to the treatment protocol. The postoperative course was uneventful. After 9 months of follow‐up, the patient is well with no clinical or radiological relapse.

## CONFLICT OF INTEREST

The authors state that there is no conflict of interest.

## AUTHOR CONTRIBUTIONS

SD: involved in drafting of manuscript, revision, collection of data, and concept design. AS, EK, and NG: involved in drafting of manuscript, revision, and collection of data. AC: involved in revision of manuscript and supervision.

## ETHICAL APPROVAL

Ethical approval was not required.
